# Rare and Complex Epilepsies from Childhood to Adulthood: Requirements for Separate Management or Scope for a Lifespan Holistic Approach?

**DOI:** 10.1007/s11910-021-01154-7

**Published:** 2021-11-24

**Authors:** Simona Balestrini, Renzo Guerrini, Sanjay M. Sisodiya

**Affiliations:** 1 Department of Clinical and Experimental Epilepsy, University College of London (UCL) Queen Square Institute of Neurology, London WC1N 3BG and Chalfont Centre for Epilepsy, London, Bucks UK; 2 Neuroscience Department, Meyer Children’s Hospital, European Reference Network ERN EpiCARE, 50139 Florence, Italy

**Keywords:** Epileptic encephalopathy, Rare syndrome, Transition, Precision medicine, Lifespan clinics

## Abstract

**Purpose:**

In this descriptive review, we describe current models of transition in rare and complex epilepsy syndromes and propose alternative approaches for more holistic management based on disease biology.

**Recent Findings:**

Previously published guidance and recommendations on transition strategies in individuals with epilepsy have not been systematically and uniformly applied. There is significant heterogeneity in models of transition/transfer of care across countries and even within the same country.

**Summary:**

We provide examples of the most severe epilepsy and related syndromes and emphasise the limited data on their outcome in adulthood. Rare and complex epilepsy syndromes have unique presentations and require high levels of expertise and multidisciplinary approach. Lifespan clinics, with no transition, but instead continuity of care from childhood to adulthood with highly specialised input from healthcare providers, may represent an alternative effective approach. Effectiveness should be measured by evaluation of quality of life for both patients and their families/caregivers.

## Introduction

Transition is the gradual process of planning, preparing, and moving from child to adult health care. This takes years of development, discussion, and implementation and should start at around 12 years of age [1] but will depend on individual circumstances and also on the availability of adult services. The transition period is characterised by a series of socio-demographically and culturally defined events and conditions, based on the natural development of each individual in human societies [2]. Transition has a biology and is underpinned by the dynamics of gene expression, throughout the body, and especially in the brain [3].

The age of majority is the gateway to adult legal status, presumptively converting legal incapacity to capacity. Today, in many countries the age of majority is eighteen, but it has historically varied from the mid-teens to the mid-twenties. Fluctuations in the age of majority have generally accorded with changes in the nature of the capabilities required of society’s adult citizens and the age by which individuals tend to attain those capabilities [4].

Transition in epilepsy has been the focus of much attention and concern [5]. Epilepsies in children have significant aetiological and phenotypic heterogeneity, and this has an impact on the transition process. For example, there are epilepsies with onset in childhood or adolescence, in individuals with relatively normal cognition, which typically remit in adolescence or later in adulthood (e.g. benign epilepsy with centrotemporal spikes or childhood absence epilepsy). However, even in drug-responsive epilepsies with no intellectual disability or any other comorbidities, issues may arise during transition, including non-adherence to medication [6] and the use of valproate in females of childbearing age [7]. Matters become further complicated in more rare and complex epilepsy syndromes, especially with comorbid intellectual disability. In these conditions, there are more barriers to accessing adult services, including the need for best interest assessments, replacement of regular follow-up appointments with as-required appointment systems, and risk for health problems being either misdiagnosed or overlooked [8]. Transition may thus become more challenging for individuals with complex epilepsy syndromes and everyone involved in their care, and it seems that in many healthcare systems around the world, the provision of a comprehensive transition programme is inadequate. Furthermore, transition in the epilepsies is burdened by the commonplace use of the term “childhood epilepsies”, with its inherent implication that some epilepsies affect only children, whilst many severe epilepsies seen in adulthood are of childhood onset but persist into adulthood. In this descriptive review, we first examine the current approaches to transition in rare and complex epilepsy syndromes. We then review the main challenges around transition in these syndromes, giving examples of the most severe ones, and finally propose alternative approaches for more holistic management with examples from other diseases.

## Methods

We searched on PubMed for articles published in English from PubMed inception to Jan 31, 2021. The following search terms were used: “epilepsy + transition”, “genetic epilepsy + transition”, “epilepsy syndrome + transition”, “epilepsy + transition model”, “lifespan clinic”, “continuity of care”, “holistic clinic”, “comprehensive health care”. Articles were also identified through searches of the authors’ own files. The search was restricted to articles published in English. The final reference list was generated based on relevance to the broad scope of this review.

## Current Approaches to Transition in Complex Epilepsy Syndromes

In the epilepsy field, transition of care is often discussed at national and international conferences. In 2016, a symposium on transition in epilepsies was held in Paris, including about 30 paediatric and adult neurologists/epileptologists from several European countries, the USA, and Canada. Three summary papers were subsequently published. [9–11] The first one described five basic themes with an important effect on transition, including brain changes in adolescence such as impulsive pleasure seeking and risky behaviours that may be the result of an imbalance between frontal and limbic maturation, puberty, sexual debut, psychological development, and bone health. Implications of these themes for transition, and some specific interventions to achieve maximum long-term adult independence and quality of life, were discussed. [9] The second paper described the outcome of childhood-onset epilepsy from adolescence to adulthood with the goal of recommending the best approach to transition. Various groups of epilepsy syndromes were discussed: disorders presenting in childhood that may have late- or adult-onset epilepsy (metabolic and mitochondrial disorders), disorders with changing problems in adulthood (tuberous sclerosis complex, Rett syndrome, Dravet syndrome, and autism), epilepsies that change with age (childhood absence epilepsy, juvenile myoclonic epilepsy, West syndrome, and Lennox-Gastaut syndrome), epilepsies that vary in symptoms and severity depending on the age of onset (autoimmune encephalitis, Rasmussen’s syndrome), epilepsies with structural aetiology that are less likely to evolve in adulthood, and childhood-onset epilepsies with a risk of later adulthood cerebrovascular disease and dementia [10]. The third paper focused on treatment issues including drugs unfamiliar to adult epilepsy specialists or without licenced indications for adults, optimisation/continuation of treatment in adulthood, treatment of comorbidities, and adherence issues [11].

Current approaches to transition for people with complex epilepsy syndromes vary. We review approaches in different countries and healthcare systems, showing that adoption of recommendations of the reports from the 2016 Paris symposium has been limited. We then consider the practical issues that need to be addressed, following up on those recommendations but also including some independent aspects.

In the UK, there are four avenues for ongoing care of young people aged 16–19 with epilepsy: remaining under paediatric services; discharge to the sole care of the general practitioner (GP); transfer to an adult neurologist or physician who does not necessarily have special expertise in epilepsy; care in a dedicated epilepsy transition service where specific adolescent and adult issues can be raised and discussed in an appropriate setting [12]. The first reported transition clinic for young people in the UK was established in 1991 in Liverpool [13].

In Italy, there are various models that slightly differ across the regional healthcare systems. In general, for drug-resistant and complex epilepsy syndromes, joint assessments by the paediatric and adult neurologist for a few visits are recommended [14]. However, it is not uncommon that for rare syndromes, continued follow-up into adulthood by the child neurologists is permitted on an individual basis in the context of a “continuity of care” plan detailing why discontinuation of a longstanding patient/team-physician relationship would be disadvantageous to the patient.

In France, at a centre for rare epilepsies in Paris, a transition programme was established in 2005, involving a single, dedicated adult neurologist taking over continuing care as young people with severe epilepsy and intellectual disability grew into adults [15]. Issues to be addressed included sexual maturation in young adults with cognitive disabilities, psychogenic seizures during stressful developmental stages in adolescence and young adulthood, and care of specific epilepsy syndromes that persist into adulthood. The process included a transfer summary from the paediatric epilepsy service plus complete access to all of the paediatric medical records. After the implementation of the transition programme, a survey for families of patients with Dravet syndrome (DS) showed success in filling the gap between paediatric and adult care systems [16]. Nevertheless, a recent survey conducted in France showed poor knowledge of published recommendations on transition in epilepsy and very rare use of a structured transition programme among paediatric and adult neurologists [17].

In Germany, a concept for a diagnosis‐independent transition programme was designed in 2008 and involved health insurance agencies at an early stage [18]. The programme started with patients with epilepsy or type 1 diabetes in the Berlin/Brandenburg region and three health insurance agencies. Three groups were delineated, according to the perceived degree of support required. The medical societies of paediatrics, internal medicine, and neurology have agreed on this programme as the basis of transition and support its use in regular health care. In 2013, the programme became a partner in the modular education system ModuS, which is offered to adolescents with chronic diseases and their parents [19].

In Canada, where there is a universal healthcare system, very few formal transition/transfer programmes exist for children moving to adult epilepsy care. A transition clinic was set up in Halifax, Nova Scotia, which was attended by a paediatric neurologist, adult epileptologist, and an adult epilepsy nurse specialist. Before the clinic appointment, the paediatric neurologist prepared a detailed written summary of the epilepsy issues, and the family prepared a written summary of the psychosocial issues. The entire team extensively reviewed these during the first visit; subsequently, there was one further follow-up visit with the same team before the final move to the adult clinic. When two of the key physicians retired from clinical practice, the structure of this clinic did not persist [20]. In Edmonton, Alberta, another epilepsy transition programme was established, run exclusively by the epilepsy nurse specialists from both paediatric and adult care. The paediatric epileptologist provided a referral letter to the adult epileptologist. The family and patient then met with the adult and paediatric epilepsy nurses and reviewed a number of key transition/transfer themes at one or more visits. Subsequently, the patient (± parents) met the adult epileptologist for ongoing care. A questionnaire assessment suggested that families strongly endorsed this process [21]. Some Canadian data about the adult outcome of children with epilepsy indicate that major social difficulties are common and not easily addressed in the adult healthcare system. A key issue, seen in a number of healthcare systems, is that children with intellectual disabilities and epilepsy are unlikely to be seen or followed by an adult neurologist [15].

There is significant heterogeneity in models of transition/transfer of care even within the same country. There is absence of evidence of the effectiveness for any specific model; neither is it known whether having a transition policy in place translates into any measurable benefit, and it seems that the main issues around transition often remain unaddressed.

There is increasing awareness that healthcare systems should introduce specific changes in order to achieve successful transition from paediatric to adult care for adolescents with chronic conditions, evaluate the short‐ and long‐term outcomes of various transition strategies, and identify those which deserve widespread implementation [22, 23].

Despite various clinical recommendations in Western countries for transition of adolescents with chronic health conditions and special needs [24–26], and some targeted efforts in the epilepsy field [9–11], we still lack a systematic strategy that is sensitive to local circumstances, healthcare systems, and cultural identities.

## The Main Challenges Around Transition in Complex Epilepsy Syndromes

Individual complex epilepsy syndromes are rare but, being numerous, collectively account for an important segment of epilepsy care. In addition, they pose particular issues not often encountered with many more common epilepsy syndromes in which typically comorbidities, in particular intellectual disability, are less prevalent and less severe.

In most secondary care hospitals across the UK, epilepsy in children and young people is managed by paediatricians with expertise in epilepsy and epilepsy nurse specialists, with selective input from tertiary-based paediatric neurologists for drug-resistant epilepsies. However, discussions around independence, sexual health, career choices, and several other aspects often do not take precedence and are perceived as inappropriate in the presence of parents [12]. In addition, during paediatric care, the young person may not have had much input from the family doctor, GP or paediatrician, or other primary care professionals, who then usually become the first point of contact for a young person in the community. This can cause the young person or the family to feel they are no longer receiving specialist care, and there may be difficulties in establishing a relationship with the primary care team in the longer term [12]. In comparison, in the adult setting the young person is likely to be seen alone within a structured consultation. They may have less frequent follow-up visits, and the onus is on the young person to seek help independently ad hoc, requiring the young person to have good knowledge of their condition and treatment and recognising when they need help [20]. These changes may have an adverse effect on the care of a young person with intellectual and other disabilities. Adult healthcare professionals may not feel adequately trained to communicate with and look after young people with complex neurological disorders and moderate to severe cognitive impairment. For individuals with cognitive disability, there are additional legal issues, the need for capacity assessment, and best interest decision-making related to clinical management and treatments. Young adults lacking capacity will need an advocate to be responsible for consenting to assessment and treatment, e.g. when inpatient admission is required and an adult ward may not be able to cater for the nuances of care in young people with complex epilepsy and disabilities, who may sometimes carry over into adulthood typical childhood features of the condition.

Treatment is often complex in rare epilepsy syndromes. Optimisation of treatment during transition should be considered, to minimise the long-term side effects of antiseizure drugs, and also before pregnancy to balance the risk of teratogenicity and seizure control [27].

**Fig. 1:**
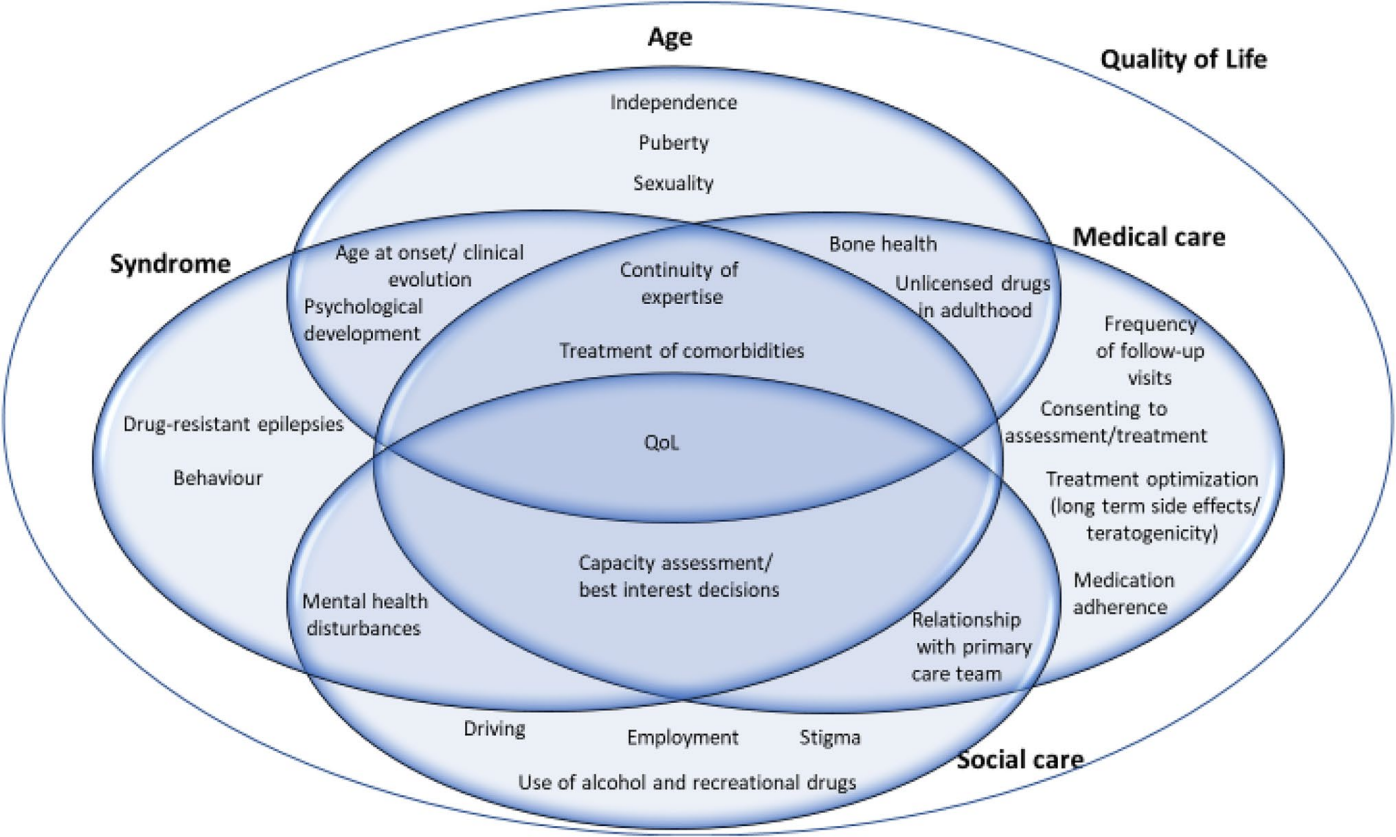
Venn diagram illustrating the main challenges around transition in complex epilepsy syndrome. The multiple issues are related with age, the specific underlying syndrome, and medical and social care needs

Based on the existing literature and on our own experience, we propose an empirical classification of the main issues around transition into four categories, with a large degree of overlap (Fig. 1): age-related (e.g. independence, puberty, sexuality, psychological development and behavioural changes, bone health), condition-related (e.g. continuity of expertise in clinical management, capacity assessment, clinical course over time, mental health disturbances, and comorbidities), medical needs (e.g. frequency of follow-up visits, consenting to assessment/treatment, treatment optimisation, use of unlicenced drugs, treatment of comorbidities, adherence issues), and social needs (e.g. driving, employment, use of alcohol and recreational drugs, psychological aspects related to independence and stigma, relationships, sexual activity). All the main issues around transition may ultimately affect quality of life. In fact, low quality of life is often described in the transition-aged population, and this is due not only to ongoing seizures, but mental health disturbances and multiple other factors may play a role [28] (Fig. 1).

The roles of families and carers need to be taken into account in chronic conditions associated with disabilities. Parents play a crucial role in transition, and they can often feel unprepared, with limited awareness of adult services, and with limited information on their children’s long-term outcomes and future needs [29, 30]. Transition can be a considerable source of parental stress, leading one review to conclude that more evidence is “needed for the development of anticipatory guidance to assist parents as their children transition into adulthood” [29]. A recent study of parental perspectives during transition has highlighted that parental roles were perceived as “time consuming, stressful, and unrelenting but necessary to protect children from harm in the face of multiple risks and uncertainties”. Conversely, protective strategies were also perceived to hinder adolescent development, family functioning, and their own development as midlife adults [31]. Recommendations were provided for working with parents and young people to manage the risks and uncertainties associated with their condition, as part of routine transitional care [31].

The most severe end of the spectrum of complex epilepsy syndromes includes early-onset developmental and epileptic encephalopathies (DEEs). These are a heterogeneous group of disorders characterised by treatment-refractory seizures and a significant degree of comorbidity including global developmental delay or regression, intellectual disability, movement disorders, autism spectrum disorders, behavioural issues [32], and often with extra-neurological involvement. The aetiology of DEEs is most often genetic, i.e. due to de novo dominant mutations, but X-linked and autosomal recessive forms can also present in neonates. There are high genetic heterogeneity and marked phenotypic pleiotropy. There are also a few exceptions with non-genetic aetiology (e.g. hypoxic-ischemic encephalopathies, postinfectious disorders). The majority of genetic diagnoses are currently made in childhood [33], whilst adults with DEEs often remain undiagnosed due to reduced access to next-generation sequencing, a long history of epilepsy and complex treatment potentially masking syndromic features, and lack of accurate information on early history [34, 35].

Data on survival and outcome in adulthood are lacking for most DEEs. From a phenotypic perspective, the recognition of an electroclinical phenotype may sometimes provide some information on clinical management and prognosis. We provide below an overview, with further details presented in Table 1, of the available prognostic data on electroclinical epilepsy syndromes which may inform the transition process.

**Table 1:** Most common electroclinical phenotypes of developmental and epileptic encephalopathies (DEEs) with a summary of available data on outcome of epilepsy and other clinical features

Electroclinical phenotype	Aetiology	Seizure onset	Clinical features	Epilepsy course	Data on outcome	References
Epilepsy of infancy with migrating focal seizures (EIMFS)	Genetic	First 6 months of life	Seizure migration between cerebral hemispheres, acquired microcephaly, cortical visual impairment, language is typically absent	Seizures increasing in frequency over the first few months, refractory to treatment	Poor outcome, profound developmental impairment and intellectual disability. Mortality is high during early childhood, typically owing to respiratory failure. In a few cases, better seizure control with less profound development delay has been reported	Burgess et al. [36] Coppola et al. [37, 38] Marsh et al. [39] Nickels & Wirrell [40] Wong-Kisiel & Nickels [41]
West syndrome (WS)	Structural, metabolic and genetic	First year of life	Epileptic spasms, hypsarrhythmia on EEG, and developmental delay or regression. Intellectual disability and autistic features are common	Epileptic spasms typically resolve by age 5 years, but other seizure types often emerge and children may develop Lennox-Gastaut syndrome (LGS).	Poor long-term prognosis although a minority of patients can achieve some degree of independence, including living independently. Aetiology is a relevant predictor of outcome	Bitton et al. [42] Camfield & Camfield [43, 44] Riikonen [45] Wong-Kisiel & Nickels [41]
Dravet syndrome (DS)	Genetic	First year of life	Initial occurrence of febrile or afebrile seizures that often evolve into status epilepticus in infants with otherwise normal early development	Epilepsy severity progressively decreases from childhood to adolescence and throughout adulthood	High incidence of premature mortality including SUDEP. Reduced occurrence of convulsive status epilepticus is associated with better seizure outcome; seizure persistence in adolescents and adults correlates strictly with cognitive and neurologic impairment; severity of intellectual disability and language impairment correlates with early onset of seizures; significant cognitive and physical disability in adulthood, including ataxia, extrapyramidal signs, crouch gait, kyphoscoliosis, dysphagia, and moderate to severe intellectual disability	Akiyama et al. [46] Catarino et al. [47] Cooper et al. [48] Darra et al. [49•] Dutton et al. [50] Dravet et al. [51] Genton et al. [52] Takayama et al. [53]
Lennox-Gastaut syndrome (LGS)	Structural, metabolic and genetic	Before the age of 8 years but later onset is possible	Triad of cognitive impairment, multiple seizure types, and slow spike and wave complexes (1.5–2.5 Hz) on the EEG. Seizure types include tonic (most common), atypical absences, atonic, and, less frequently, myoclonic and focal onset seizures	Infants can first develop West syndrome and later evolve into LGS. Seizures persist into adulthood in more than 90% of patients	The rate of premature death is estimated at 5–17% during follow-ups of 12–21 years, with up to half cases dying of complications of seizures. Seizure and cognitive impairment present in adulthood	Arzimanoglou et al. [54] Commission on Classification and Terminology of the International League Against Epilepsy [55] Kim et al. [56] Kerr et al. [57] Montouris [58] Nickels et al. [32] VanStraten & Ng [59]
Continuous spikes and waves during sleep (CSWS)/Landau-Kleffner syndrome (LKS)	Structural, metabolic and genetic	Between 2 and 12 years	Epilepsy, neurocognitive regression, EEG pattern of electrical status epilepticus during sleep (ESES)/acquired aphasia is the core symptom in LKS	In ESES, remission of seizures in adolescence is seen in almost all patients	Poor cognitive outcome in > 50% cases/severe and disabling language disturbances in adulthood in LKS	Duran et al. [60] Kramer et al. [61] Liukkonen et al. [62] Praline et al. [63] Tassinari et al. [64]

Understanding the natural history of disease and identification of prognostic factors are crucial to predict long-term outcomes, to adopt preventative strategies, and to develop disease-modifying treatments. However, our knowledge is still very limited especially in relation to outcome of rare genetic epilepsy syndromes. We can only speculate that multiple factors contribute to disease course, including brain development, specific gene abnormality and associated neurophysiological dysfunction, changes in gene expression over time, role of modifier genes, and also clinical factors such as seizure frequency and behavioural disturbances.[65••] We note that the latter do not always show a linear correlation with outcome; e.g. in DS, the outcome in adulthood is generally poor although epilepsy severity and behavioural abnormalities tend to decrease over time.[49•]

With the advent of next-generation sequencing, more and more gene mutations are being identified as the cause of DEEs and other complex epilepsy syndromes. As a consequence, we are hoping to switch from a syndrome-based description of clinical phenomenology to a more specific understanding based on pathophysiology. Likewise, empirical treatment approach based on the electroclinical phenotype will likely be gradually substituted by an algorithm-based treatment approach aiming to reverse or inhibit the neurophysiological dysfunction caused by the genetic defect [66]. However, such precision medicine treatments are currently available only for a minority of genetic epilepsies, and most patients are still treated on a trial-and-error process. Equally, our understanding of pathophysiological mechanisms and disease course remains very limited for many rare genetic epilepsy syndromes, where our current clinical practice is still mostly grounded on electroclinical phenotypes. The latter are much less defined in adulthood, and in general the concept of DEE/EE is generally less well-defined in older patients. In fact, most studies investigating clinical features and treatment response rely on paediatric data, although obviously appropriate treatment strategies are also required for adults where management becomes even more complicated due to comorbidities and disease evolution.

Identification of a genetic diagnosis may lead to treatment changes with subsequent clinical improvement at any age, for example, withdrawal of sodium channel blockers in DS [47] or creatine supplementation in cerebral creating deficiency type I. [67] However, there are individuals where the causative role of gene mutation is more difficult to establish, [68] or where the putative “precision treatment” is ineffective [69•], or where there are no direct treatment implications following genetic diagnosis.

Among the multiple factors that may play a role in the natural history of complex genetic epilepsy syndromes, gene expression and epigenetic mechanisms probably feature. For some genes, we know that they continue to be highly expressed in the adult human brain, but we do not know how this relates to clinical outcome [70]. There are some suggestions that differential developmental expression pattern of sodium channel genes may influence age at clinical onset and disease course [69•, 71]. To our knowledge, there are no data on the influence of epigenetic mechanisms on the course of complex epilepsy syndromes.

Overall, these examples of genetic disorders with phenotypic heterogeneity and widely variable age at onset provide a strong indication that clinical discontinuity of care between paediatric and adult medicine, although to some extent unavoidable in many current health systems, would result in oversimplified approaches and detrimental effects on patient care.

## Lifespan Clinics as an Alternative Healthcare Model — Examples from Other Diseases

The current care transition models and recommendations do not address the complexity of rare genetic epilepsy syndromes. There is significant heterogeneity across various transition strategies, and many factors are not taken into account, e.g. the impact of social and cultural environment on chronic health conditions [72]. The main weaknesses of existing models include absence of records of early history; lack of expertise in complex conditions such as genetic DEEs across adult care services [73–75]; late age at the start of transition programmes [75]; and reduced access to new treatments for adult patients with rare conditions (e.g. clinical trials, drugs licenced only for paediatric treatment, target population for experimental treatment trials, etc.). Furthermore, there are specific aspects of chronic epilepsy syndromes in adults that require dedicated expertise, such as physiological somatic changes in adulthood, ageing, and other organ involvement becoming symptomatic in adulthood.

Lifespan clinics, providing follow-up from disease onset throughout life including childhood and adulthood, are alternative models to manage rare and complex conditions. Examples of a similar approach exist in other chronic health conditions. The medical community has recognised that specialised comprehensive care centres can decrease the morbidity and mortality associated with certain chronic diseases. Reduced hospitalisation, morbidity, and mortality rates have followed with commissioning of specialised centres with multidisciplinary teams for haemophilia and cystic fibrosis [76, 77]. Such approaches with holistic management have also been advocated in complex genetic syndromes such as Tuberous Sclerosis Complex (TSC) [78], where, for example, consensus recommendations on surveillance and management are relevant to the entire lifespan [79]. Lifespan care is already provided by clinical genetics services, often including multidisciplinary team–based approaches [80]. There are also reference centres, physical and virtual, for rare disease [81], including the European Reference Network for Rare Neurological Diseases (ERN-RND) which was launched by the European Union to support patients and families affected by rare neurological diseases (RND) and provide specialised knowledge, treatment, and resources. The ERN for rare and complex epilepsies (EpiCARE) includes highly specialised health centres in 24 European countries with expertise in such epilepsies and is organised in work packages and modules that approach clinical and research areas of epilepsy in their entirety as a whole, irrespective of age-related issues, with the only exception for neonatal seizures [82].

Given the unique presentations and intricacy of rare and complex epilepsy syndromes in which epilepsy is the main clinical element, we propose here the adoption of lifespan clinics where there is no transition but continuity of care from childhood to adulthood with multidisciplinary and highly specialised input from healthcare providers. The specialist involved should have extensive knowledge of principles of the disease and of existing precision medicine strategies for each rare condition, with facilitated access to treatment options and innovative therapies. The epilepsy nurse specialist, currently in existence only in some countries, could play a key role in lifespan management and in communication with family, primary care providers, and other local services (e.g. pharmacies, social services, rehabilitation centres). This model would also facilitate more comprehensive research programmes on rare diseases and address transition with a more individualised approach without a fixed age for care transfer. Such personalised approaches should aim to a better understanding of the underlying pathophysiological dysfunction, including its association with brain maturation and age-related changes in each specific genetic condition. Multiple barriers could arise in implementing lifespan clinics, including restructuring of medical training and healthcare delivery, variation of regulations across different countries, and lack of expertise bridging paediatric and adult care. New professional roles would be needed bridging childhood course with adult outcome, with appropriate knowledge, skills, attitudes, and organisational support to serve people with disabilities, including expertise in assessing the needs and options for assistive technology, general-use technologies, and home modifications. As rare and complex epilepsy syndromes may require health care from early life to the geriatric phase, multidisciplinary teams will be required, and our holistic approach proposal may be implemented in a stepwise manner starting with patients with the most difficult-to-treat epilepsies. Although this approach would require substantial changes in healthcare provision, there are clear advantages and strengths that would justify its implementation in clinical practice.

## Conclusions

Transition describes “a purposeful, planned process that addresses the medical, psychosocial and educational/ vocational needs of adolescents and young adults with chronic physical and medical conditions as they move from child-centred to adult-oriented health systems” [83]. Current models for transition in epilepsy syndromes are heterogeneous and overall do not seem to be highly effective in addressing the complexity of rare genetic conditions. There are a high risk of adolescents, young adults, or even older adults with intellectual disability, whose clinical presentation maintains characteristics usually seen in the paediatric age, remaining in a grey area with inadequate healthcare provision and subsequent higher risks of morbidity and mortality in adulthood. Lifespan clinics could represent a feasible and effective option for specific rare, complex epilepsy syndromes requiring high level of expertise and multidisciplinary approach. Effectiveness should be measured by reduced rate of morbidity and mortality and improved quality of life for both patients and families/caregivers. We propose lifespan in alternative to transition clinics as an additional route to promote precision medicine in rare and complex epilepsies [84].
